# Influence of artificial diets on biological characteristics and digestive enzymes of *Coccinella septempunctata* L.

**DOI:** 10.1093/jisesa/iead022

**Published:** 2023-06-03

**Authors:** Ying Cheng, Yi Yu, Wenhong Li, Fengliang Li

**Affiliations:** Institute of Plant Protection, Guizhou Provincial Academy of Agricultural Sciences, Guizhou, Guiyang 550006, China; Institute of Entomology, Guizhou University/Guizhou Provincial Key Laboratory for Agricultural Pest Management of the Mountainous Region, Guizhou, Guiyang 550025, China; Institute of Plant Protection, Guizhou Provincial Academy of Agricultural Sciences, Guizhou, Guiyang 550006, China; Institute of Plant Protection, Guizhou Provincial Academy of Agricultural Sciences, Guizhou, Guiyang 550006, China

**Keywords:** Coccinella septempunctata, artificial diet, biological characteristics, digestive enzyme

## Abstract

In order to improve the rearing of *Coccinella septempunctata* L. (Coleoptera: Coccinellidae), nutrients such as shrimp, pollen, honey, and lard were added to the basic artificial diet, and the effects of the artificial diet on biological parameters and digestive enzymes were evaluated. The results show that beetles feeding on the supplemented diet exhibited pupation, emergence, fecundity, and hatching rates that were 102.69%, 125.02%, 162.33%, and 119.90% of those supplied with the basic diet, respectively. The addition of shrimp and pollen to the basal diet improved protease, trypsin, chymotrypsin, and aminopeptidase activity in larvae and female adults. The addition of lard improved lipase activity in female adults, and the addition of honey improved invertase activity in adults of both sexes. This study provides guidance for improving the nutritional benefits of ladybug artificial diets.

Investigators began experimenting with the formulation of ladybug artificial diets in the 1950s (Singh 1997). In 1958, Smirnoff 1958 formulated an artificial diet that contained prey powder as the primary ingredient, and the diet was successful in rearing multiple species of predatory ladybugs. Investigators then began focusing on ladybug artificial diets containing components that did not contain prey or insect ingredients. More recently, researchers have investigated artificial diets for a variety of predatory ladybirds, including *Harmonia axyridis* (Berkvens et al. 2008, Ali et al. 2016), *Coccinella septempunctata* (Singh et al. 2014, Wu et al. 2020, Cheng et al. 2022), *Propylea japonica* (Zhang et al. 2007, Wang et al. 2008) and *Adalia bipunctata* (Bonte et al. 2010). Variation in the formulation of artificial diets were shown to affect the length of the ladybug larval stage, body weight, pupation, emergence, and fertility.

Insects need carbohydrates, proteins, lipids, sterols, vitamins, inorganic salts, and other nutrients to meet requirements for growth, development, and reproduction (Zeng and Chen 2009). If the content and proportion of nutrients in the artificial diet are suitable for their metabolic needs, insects grow and develop normally and have high fecundity (Thompson 1999, Cohen et al. 2005, Su et al. 2014). Insects need different kinds and quantities of nutrients in different growth and developmental stages, and different proteases are required depending on the protein source and developmental stage (Zeng and Cohen 2000, Pauchet et al. 2010). Trypsin, chymotrypsin, aminopeptidase, lipase, and invertase play important roles in insect digestion, growth, and development (Zeng et al. 2019). We previously compared the nutritional composition of basic artificial diets with the *Aphis craccivora* diet and found that the fat and sugar content in the artificial diet was lower than *A. craccivora*, whereas the protein and amino acid content in the artificial diet was higher than *A. craccivora* (Cheng 2018). However, transcriptomic analysis of *C. septempunctata* fed on the artificial diet revealed that differentially expressed genes in amino acid metabolism were generally downregulated, indicating that the absorption and utilization of proteins and amino acids in the artificial diet was poor, thus impacting growth and development (Cheng et al. 2020). Consequently, it is critical to consider the conversion and utilization of dietary ingredients by ladybugs and include natural compounds that are nutrient-rich. We added various natural nutrient sources into the basic artificial diet as single factors and found that shrimp, pollen, lard, and honey could increase body weight, fecundity, and the egg hatching rate of ladybirds (Yu et al. 2022). In the current study, shrimp, pollen, honey, and lard were added to the basic artificial diet, and developmental indicators and enzyme activities (e.g., trypsin, chymotrypsin, aminopeptidase, lipase, and invertase) were compared for ladybugs supplied with basic, supplemented, and aphid diets (*A. craccivora*). The results illustrate the effects of supplemented diets on biological characteristics, digestion, and effects on several enzymes involved in the *C. septempunctata*.

## Materials and Methods

### Insects

Two hundred *C. septempunctata* adults were collected from wheat fields located at the Guizhou Provincial Academy of Agricultural Sciences, Guiyang, China; eggs obtained from the adults were used in experiments. Adults were fed on *A. craccivora*, egg masses were collected twice daily, and hatched larvae were fed on *A. craccivora* as described (Cheng et al. 2018). The experiments were conducted in growth chambers at 70 ± 5% RH, 25 ± 1^o^C with a 16: 8 h light: dark photoperiod.

### Artificial Diet Preparation

The basic diet ingredients were previously reported (Cheng et al. 2018) and adjusted in the present study as follows: milk powder, 15 g (2.54% weight); pork liver, 105 g (17.81% weight); olive oil, 2 g (0.34% weight); sucrose, 45 g (7.63% weight); corn oil, 2 g (0.34% weight); eggs, 15 g (2.54% weight); powdered yeast, 5 g (0.85% weight); casein, 5 g (0.85% weight); cholesterol, 0.5 g (0.08% weight); protein powder, 4.5 g (0.76% weight); vitamin E, 0.75 g (0.13% weight); vitamin C, 1 g (0.17% weight); honey, 7.5 g (1.27% weight); agar, 6.25 g (1.06% weight); distilled water, 375 g (63.61% weight); and 65% juvenile hormone III, 3 μl (omitted from larval diets). The supplemented diet contained the ingredients in the basic diet and was amended with 72 g (10.24% weight) shrimp, 12 g (1.71% weight) pollen, 14.5 g (2.06% weight) honey, and 15 g (2.13% weight) lard. A tissue triturator was used to grind the pork liver into a paste. With the exception of agar and sterile water, all ingredients were weighed and mixed together. Sterile water and agar were combined, heated to dissolve, and cooled to 40–50°C. The agar-water suspension was then combined with the other ingredients, stirred, cooled to room temperature, and stored at 4°C.

### Feeding Protocols

Sixty 1st instar *C. septempunctata* larvae were used in each treatment. Individual larvae were transferred to petri plates and supplied with diets (0.02 g) and water using established protocols (Yu et al. 2022). Larval survival and development were recorded daily until emergence. Twenty larvae were considered as 1 replication, and 3 replications were used in each treatment.

The pupation and eclosion rates of *C. septempunctata* larvae fed on basal and supplemented diets were low, and eclosed adults were small and unable to produce eggs. Therefore, the adult test insects for feeding artificial diet were obtained by feeding larvae with *A. craccivora*. After a 24 h eclosion period, adults obtained from the *A. craccivora* diet were paired. Ten pairs were considered as 1 replication, and 5 replications were used in each experiment. Paired adults were transferred into 500 ml plastic bottles and provided with artificial diet (0.04 g), which was changed daily. Relative humidity was maintained with moist cotton, and perforated bottle caps provided ventilation. A piece of paper (5 × 10 cm) was positioned inside the bottle as an oviposition site as described (Cheng et al. 2018). The papers containing eggs were collected twice daily and placed in Petri dishes for hatching. This process continued for 50 days, and pre-oviposition, fecundity, hatching rates, and survival rates of female adults were recorded.

The control group was fed with *A. craccivora* during the larval and adult stages. The feeding methods and repetitions of larvae and adults in the control group were identical to insects supplied with the basal and supplemented diets. The *C. septempunctata* control group was provided with sufficient numbers of aphids for daily feeding. All experiments were conducted in growth chambers at 70 ± 5% RH, 25 ± 1°C, and a 16: 8 h light: dark photoperiod.

### Determination of Enzyme Activity

Fourth instar larvae and adults (20 days after emergence) were starved for 12 h and transferred to a −20°C freezer. Test kits for total protease, trypsin, chymotrypsin, aminopeptidase, invertase, and lipase were purchased from Shanghai Best Choice Biotechnology Co., Ltd.

### Protease Activity

Test insects (0.1 g) were homogenized in an ice bath at a 1:10 ratio of insect weight vs. 0.1 mM PBS buffer and centrifuged at 4°C for 10 min at 10,000 rpm/min. The supernatant containing the crude enzyme solution was placed on ice for testing. Total protease activity was assayed according to the kit instructions, and absorption was measured at 680 nm using a microplate reader, and the unit of total protease activity is U/g.

### Trypsin Activity

Insects (0.1 g) were homogenized in an ice bath at a 1:10 ratio of insect weight vs. 0.02% phenol red plus 1 mol/liter sodium hydroxide solution, and centrifuged at 4°C for 10 min at 8,000 rpm/min. Supernatants containing crude enzyme extracts were incubated on ice prior to testing, and trypsin activity was assayed according to the kit instructions. Absorption was measured at 253 nm on a microplate reader, and the unit of trypsin activity is U/g.

### Chymotrypsin Activity

Test insects (0.1 g) were homogenized in an ice bath at a 1:10 ratio of insect weight vs. 0.0037% hydrochloric acid solution, and centrifuged at 4°C for 10 min at 8,000 rpm/min. Supernatants containing crude enzyme extracts were incubated on ice, and chymotrypsin activity assays were performed according to the kit instructions. Absorbance was measured at 237 nm using a microplate reader, and the unit of chymotrypsin activity is U/g.

### Aminopeptidase Activity

Insects (0.1 g) were homogenized in an ice bath at a 1:10 ratio of insect weight vs. 0.1 mM PBS buffer, and centrifuged at 4°C for 10 min at 8,000 rpm/min. Supernatants were placed on ice and aminopeptidase assays were performed according to the kit instructions. Absorbance was measured at 405 nm, and the unit of aminopeptidase activity is nmol/min/g.

### Lipase Activity

Insects (0.1 g) were homogenized in an ice bath at a 1:10 ratio of insect weight vs. 0.1 mM PBS buffer, and centrifuged at 4°C for 30 min at 15,000 rpm/min. Supernatants were placed on ice, and lipase assays were performed as recommended by the manufacturer. Lipase activity was detected using olive oil as a substrate, and hydrolysis of the substrate was detected by measuring absorbance at 710 nm on a microplate reader. The unit of lipase activity is U/g.

### Invertase Activity

Insects (0.1 g) were homogenized in an ice bath at a 1:10 ratio of insect weight vs. 100 mM Tris HCl (pH 7.0), and centrifuged at 4°C for 10 min at 8,000 rpm/min. Supernatants were placed on ice, and invertase assays were performed as recommended by the manufacturer. Sucrose was used as a substrate, and hydrolysis was measured at 520 nm. One microgram of sucrose per gram of insect tissue was used to define 1 U of invertase activity, which was calculated for 1 min; invertase activity was expressed in μg/min/g.

### Statistical Analysis

Fisher’s least significant difference (LSD) test (*P* ≤ 0.05), one-way analysis of variance (ANOVA), and DPS v. 17.10 were used to compare biological characteristics and enzyme activity in ladybugs fed with the basic, improved, and aphid diets. The data were not transformed prior to analysis, and the results are shown as means ± standard error (SE). Figures showing enzyme activity were prepared in Microsoft Excel 2007.

## Results

### Effects of Artificial Diets on Biological Characteristics of Larvae

Growth and development parameters for *C. septempunctata* larvae varied with the basal, supplemented, and aphid diets (Table 1). Development time for *C. septempunctata* reared on the basic and supplemented diets was longer than on the aphid diet, and the difference was significant (df = 2,8, *F* = 10.7390, *P* = 0.0246). The 4th larvae weight for *C. septempunctata* reared on the basic and supplemented diets was significantly lighter than on the aphid diet (df = 2,8, *F* = 0.0220, *P* = 0.0010). On the supplemented diet, the survival rate of larvae was lower than that on the basic and aphid diets (df = 2,8, *F* = 0.2060, *P* = 0.8220). The pupation rates of *C. septempunctata* reared on the basic and supplemented diets were significantly lower than those reared on aphids (df = 2,8, *F* = 7.4150, *P* = 0.0451). The emergence rates of *C. septempunctata* reared on the basic and supplemented diets were also significantly lower than those reared on aphids (df = 2,8, *F* = 25.9550, *P* = 0.0051), and the rates were higher on improved vs. basic diets (df = 2,8, *F* = 1.3160, *P* = 0.3701). Overall, development, survival, pupation, and emergence were highest when ladybug larvae were supplied with an aphid diet.

**Table 1.** Biological parameters for *Coccinella septempunctata* larvae supplied with different diets

Treatment	Developmental time (d)	4th larvae weight (mg)	Survival (%)	Pupation (%)	Emergence (%)
Basic diet	13.10 ± 0.36 a	27.32 ± 1.06 b	86.67 ± 2.42 a	61.67 ± 6.11 b	33.33 ± 4.01 b
Supplemented diet	12.83 ± 0.25 a	28.34 ± 0.46 b	85.00 ± 4.01 a	63.33 ± 3.38 b	41.67 ± 3.89 b
Aphid diet	11.17 ± 0.12 b	40.20 ± 0.61 a	90.00 ± 6.41 a	88.33 ± 3.38 a	76.67 ± 3.09 a

Data represent means ± SE. Values with different lowercase letters in the same column indicate significance at *P* ≤ 0.05.

### Effects of Artificial Diets on Biological Characteristics of Adults

The weight gain of female ladybugs fed on supplemented diets was higher than insects supplied with the basic and aphid diet (df = 2,8, *F* = 1.7300, *P* = 0.2874); furthermore, the weight gain of male ladybugs fed on supplemented diets was higher than weight gain on the basic and aphid diet (df = 2,8, *F* = 1.2000, *P* = 0.2894) (Table 2). The survival rates of female ladybugs fed on supplemented diets were higher than the basic diet (df = 2,8, *F* = 3.3394, *P* = 0.0882) (Table 2). The length of the pre-oviposition period was shorter for females fed on the basic and modified diets as compared to the aphid diet but the difference was not significant (df = 2,8, *F* = 1.2670, *P* = 0.3326). The fecundity of ladybugs fed on supplemented diets was significantly higher than the basic diet and significantly lower than the aphid diet (df = 2,8, *F* = 36.1710, *P* = 0.0001). The hatching rates of ladybugs fed on supplemented diets were significantly higher than the basic diet, and were significantly lower than the aphid diet (df = 2,8, *F* = 23.1950, *P* = 0.0005).

**Table 2.** Biological parameters for *Coccinella septempunctata* adults supplied with different diets

Treatment	10-day weight gain of female(mg)	10-day weight gain of male (mg)	50-day female survival rate (%)	Preoviposition (days)	Total fecundity (no.)	Hatching rate (%)
Basic diet	10.54 ± 0.36 b	9.25 ± 0.08 a	44.00 ± 5.81 b	10.36 ± 0.43 a	283.12 ± 21.79 c	50.56 ± 2.19 c
Supplemented diet	13.36 ± 0.84 a	10.49 ± 0.51 a	62.00 ± 1.33 ab	10.28 ± 0.24 a	459.60 ± 20.18 b	60.62 ± 1.91 b
Aphid diet	12.42 ± 0.32 a	9.96 ± 0.43 a	64.00 ± 6.09 a	11.12 ± 0.31 a	676.61 ± 35.56 a	72.20 ± 1.29 a

Data represent means ± SE. Values with different lowercase letters in the same column indicate significance at *P* ≤ 0.05.

### Effects of Artificial Diets on Digestive Enzymes in Ladybird Beetles

**Fig. 1.**
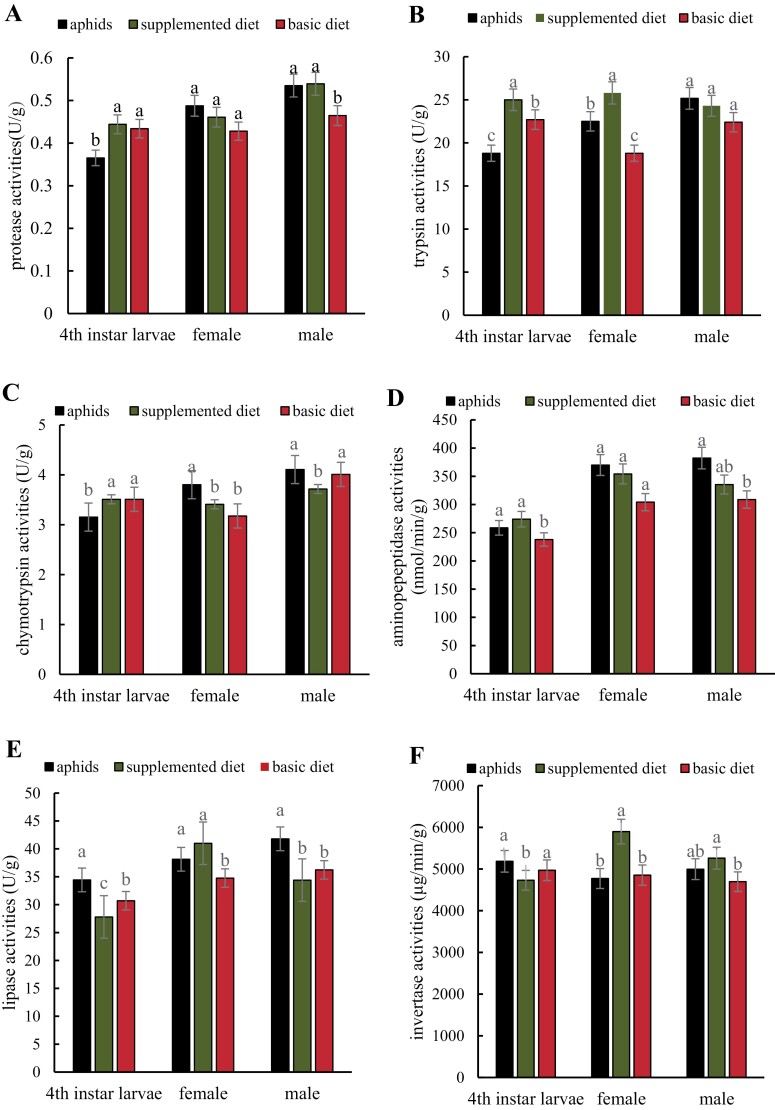
Enzyme activity in *Coccinella septempunctata* feeding on basic, improved, and aphid diets. Panels show activity for protease (A), trypsin (B), chymotrypsin (C), aminopeptidase (D), lipase (E), and invertase (F). The significance of difference is indicated by *P* ≤ 0.05, the *P* value across only each group of 3 bars. The results are presented as mean ± SE.

Fourth instar larvae feeding on artificial diets had significantly higher protease activity than those feeding on aphids (df = 2,8, *F*= 28.8660, *P* = 0.0042) (Fig. 1A). There was no significant difference in protease activity among female adults feeding on artificial and aphid diets (df = 2,8, *F*= 3.7870, *P* = 0.1195). When compared with the basic diet, protease activity was significantly higher for male adults reared on aphids and supplemented diets (df = 2,8, *F*=24.2170, *P* = 0.0058). Overall, protease activity was higher in male adults than 4th instar larvae and female adults.

Trypsin activity in 4th instar larvae fed on the supplemented artificial diet was significantly greater than the basic and aphid and basic (df = 2,8, *F*= 99.5260, *P* = 0.0004) (Fig. 1B). When female adults were fed on the supplemented diet, trypsin activity was significantly greater than the aphid and basal diets (df = 2,8, *F*= 51.4780, *P* = 0.0014), and the latter diet resulted in the lowest trypsin activity. Trypsin activity was higher when male adults were supplied with aphids, but the difference was not significant when compared to the supplemented and basic diets (df = 2,8, *F* = 3.1000, *P* = 0.1538).

Chymotrypsin activity was significantly higher when 4th instar larvae were supplied with the basal and supplemented diets as compared to the aphid diet (df = 2,8, *F* = 15.1480, *P* = 0.0136) (Fig. 1C). When adult females were fed on the aphid diet, chymotrypsin was significantly higher than the improved and basic diets (df = 2,8, *F* = 10.4320, *P* = 0.0259). In adult males, chymotrypsin activity was significantly lower on the supplemented diet vs. the basic and aphid diets (df = 2,8, *F*= 8.7720, *P*= 0.0345).

Aminopeptidase activity in 4th instar larvae fed on the supplemented diet was higher than activity in larvae feeding on the aphid and basal diets (df = 2,8, *F* = 21.4510, *P* = 0.0073) (Fig. 1D). In adult females, aminopeptidase was less active on the basic diet vs. the aphid and supplemented diets but the difference was not significant (df = 2,8, *F*= 3.3380, *P* = 0.1404). In adult males, aminopeptidase activity was lower with the basic diet and was significantly higher on the aphid diet (df = 2,8, *F*= 7.3330, *P* = 0.0459). Overall, aminopeptidase activity in male and female beetles was higher than in larvae.

Fourth instar larvae feeding on the modified and basal diets had significantly lower lipase activities than those feeding on the aphid diet (df = 2,8, *F* = 397.2650, *P* = 0.0000) (Fig. 1E). Furthermore, male adults feeding on the modified and basal diets had significantly lower lipase activities than those feeding on the aphid diet (df = 2,8, *F* = 46.3860, *P* = 0.0017). The lipase activity of female adults supplied with the supplemented diet was significantly higher than females feeding on aphids or the basal diet (df = 2,8, *F* = 14.8970, *P* = 0.0140).

Invertase activity in 4th instar larvae supplied with the supplemented diets was lower than activity on the basic and aphid diet (df = 2,8, *F* = 15.3630, *P* = 0.0133) (Fig. 1F). In female adults, invertase activity was significantly higher when ladybugs were supplied with the supplemented diet vs. the aphid and basal diets (df = 2,8, *F* = 79.6410, *P* = 0.0006). Similarly, invertase activity was significantly higher in male adults supplied with the supplemented diet as compared to the basic diet (df = 2,8, *F* = 12.9030, *P* = 0.0180).

## Discussion

Artificial diets for ladybugs can be divided into insect-derived and non-insect-derived categories; in the former, nutrients can be selected to closely resemble prey insects. Insect-derived diets are commonly used to rear ladybugs but the nutrients can be costly and difficult to obtain. The rearing of *C. septempunctata* began with artificial diets containing insect materials, and this was followed by including aphids, *Trichogrammatid* pupae, and *Ephestiakuehniella* eggs (Cao and Zhang 1994, Sun et al. 2001, Zhou et al. 2017). There are numerous reports on the use of non-insect materials for ladybird diets. *C. septempunctata* was first reared on a pork liver diet in 1977, and this was subsequently amended with different sugars, lipids, amino acids, olive oil, and juvenile hormone analogs. Sucrose was shown to promote feeding and significantly increased the amount and rate of oviposition; however, the addition of various amino acids failed to improve the reproductive ability of ladybugs (Fu and Chen 1982). The addition of juvenile hormone analogs and olive oil increased the oviposition rate and adult body weights, shortened the pre-oviposition period, stimulated feeding, and improved food intake and conversion (Chen et al. 1984). When ladybugs were reared on the basic diet in this study, larval pupation and emergence rates were 61.67% and 33.33%, respectively (Table 1), whereas adult fecundity and hatching rates were 283.12 eggs and 50.56% (Table 2), respectively. With the supplemented diet, the pupation and emergence rates of larvae were 63.33% and 41.67%, whereas the fecundity and hatching rates of adults were 459.60 eggs and 60.62%, respectively. These results indicate significant improvement of the adult basal diet when amended with shrimp, pollen, honey, and lard; however, the biological indices of larvae fed with the supplemented diet did not improve significantly, which may be caused by the high amount of lard added to the basic diet. In our previous experiments, the addition of lard to the basic diet resulted in decreased larval survival (Yu et al. 2022). In subsequent experiments, we showed that the amount of supplemental lard should be reduced when optimizing the content of other components (Cheng et al. 2022).

Protein, fat, and sugar are crucial nutrients in insect artificial diets (Zeng and Chen 2009). The digestion and utilization of proteins and fats determine the development and reproduction of insects, especially carnivorous species. Protease and lipase are the main digestive enzymes of carnivorous insects, and protease can rapidly degrade proteins in prey tissues into amino acids and peptides, thus supplying nutrients for the development and reproduction of carnivorous species (Agusti and Cohen 2000, Zeng and Cohen 2000, Boyd et al. 2002). In this study, protease, trypsin, and chymotrypsin activities in larvae fed on the basic and supplemented diets were significantly higher than those fed on aphids, and the enzyme activities in the supplemented diet were generally higher than the basic diet. In addition, shrimp are rich in protein, and the protease activity of ladybug larvae was elevated after shrimp was added to the basic diet; this result suggests that the added shrimp was beneficial to absorption and utilization of nutrients in response to the requirements for protein in the ladybug larvae. The development time, survival rate, and pupation rate were not different between the shrimp-included diet and basic diet, possibly because the insects have to produce more enzymes to get their metabolic needs in the shrimp-included diet. Aminopeptidase activity in larvae fed with the supplemented diet was higher than that obtained with the basic and aphid diets, which may be attributed to the rich nutrients provided by pollen and sufficient quantity. However, the aminopeptidase activity of adults fed with the supplemented diet was lower than adults fed with the aphid diet; this may be attributed to the insufficient amount of pollen in the basic diet. The lipase activity of larvae and male adults fed on the supplemented diet was lower than the aphid and basic diets. This reduced lipase activity could have occurred because the additional lard in the basal diet is not conducive to decomposition and utilization by ladybird larvae and male adults. Lipase activity in female adults fed on the supplemented diet was higher than the aphid and basic diets; this might have occurred because female adults require more fat for reproduction, and the supplemented fat in the basic diet was enough to meet the needs of female adults.

The invertase activity of larvae fed on the amended diet was lower than invertase activity in larvae supplied with the aphid and basic diets, indicating that excessive levels of honey probably were not conducive to larval absorption or digestion of nutrients. Furthermore, the invertase activity of adults fed on the amended diet was higher than invertase activity in adults supplied with the aphid and basic diets. Thus, we concluded that the honey content in the amended diet met the nutritional needs of adults.

In this study, female adults supplied with the supplemented diet had higher protease, trypsin, chymotrypsin, aminopeptidase, lipase, and invertase activity than those supplied with the basic diet; furthermore, trypsin, lipase, and invertase activity were higher than levels obtained with the aphid diet. Collectively, these results suggest that adding natural nutrients such as shrimp, pollen, honey, and lard to the basic diet was suitable for the absorption and utilization needs of female adults and met their basic nutritional requirements. Protease, trypsin, aminopeptidase, and invertase activities of male adults fed on the supplemented diet were higher than those fed on the basic diet, indicating that the added shrimp, pollen, and honey were also suitable for male adult ladybugs.

To summarize, amending the basic diet with shrimp, pollen, honey, and lard improved pupation and emergence rates of larvae and increased egg production and hatching rates of adults. The addition of shrimp, pollen, and honey to the basal diet can improve protease, trypsin, chymotrypsin, and aminopeptidase activity in larvae and female adults. The addition of lard elevated lipase activity in female adults but decreased activity in larvae and male adults. Increasing honey content in the basic diet was not conducive to larval digestion and utilization. These results provide a theoretical basis for further optimization of ladybug artificial diets. The results show that protease, trypsin, chymotrypsin, aminopeptidase, lipase, and invertase activities of ladybugs reared on the amended diet were significantly different from enzyme activities of ladybugs reared on the aphid diet. The current study shows that the activity of digestive enzymes is affected by multiple factors and warrants further study. Furthermore, the protein, lipid, and carbohydrate content of the artificial diet needs to be further modified and evaluated.
